# A Case of Darier Disease Treated With a Dye Laser

**DOI:** 10.1111/srt.70057

**Published:** 2024-10-04

**Authors:** Mario Sannino, Beatrice Marina Pennati, Tiziano Zingoni, Irene Fusco, Giovanni Cannarozzo, Elena Campione, Giuseppe Lodi

**Affiliations:** ^1^ Lasers in Dermatology Unit University of Rome Tor Vergata Rome Italy; ^2^ El.En. Group Calenzano Italy; ^3^ Unit of Dermatology University of Campania “Luigi Vanvitelli” Naples Italy

Dear Editor,

Darier disease (DD), also known as keratosis follicularis, is a genodermatosis that is autosomal dominant‐inherited [1]. DD affects men more often than women, but it is an uncommon disease with an incidence of 1 case per 100 000 persons. DD is caused by a mutation in the ATP2A2 gene [1]. Sarcoendoplasmic reticulum calcium ATPase (SERCA2), a calcium pump found in the endoplasmic reticulum (ER), is encoded by this gene. To process junctional proteins like desmogleins and desmoplakins, the ER needs a high concentration of calcium, which is carried there by this pump, which moves calcium from the cytosol inside the reticulum. Poor keratinocyte cohesion and abnormal junctional protein processing result from impaired SERCA2 function. It is thought that this deficiency results in acantholysis, a cellular stress response that is defined as the loss of link between keratinocytes [2].

Brown dyskeratotic papular eruptions are DD's defining feature, with a preference for seborrheic and frictionless body regions. Patients may experience social isolation because of their itching, pain, burning feeling, or subsequent illness with odor. Heat, humidity, friction, infection, and exposure to UV light can all cause or worsen flares and their progression. As a consequence, DD patients present a strong susceptibility to significant bacterial and viral cutaneous infections, such as human papillomavirus, herpes simplex virus, and poxvirus infections [2].

Although many traditional therapies have been described in the literature, it is still challenging to achieve a complete remission of the illness. Topical retinoic acid, vitamin D derivatives, 5‐fluorouracil, and nonsteroidal anti‐inflammatory medications (such as diclofenac sodium gel) can be used to treat patients with mild skin lesions. Nonetheless, there is not enough existing scientific data to conclude that these treatments are fully effective, especially in more severe instances. Different types of lasers have been notified as alternative treatments, such as CO_2_ and Er:YAG lasers, pulse dye lasers (PDL), and diode lasers [3, 4]. CO_2_ lasers and PDL are most commonly used but CO_2_ and Er:YAG usually are responsible for the most frequent and severe side effects like discomfort, dyspigmentation, burning, edema, and erythema following the treatment.

With these premises, we present a 65‐year‐old male case of DD with extended and typical keratotic and eroded skin lesions in the axillary area. He was treated with a dye laser device (SYNCHRO VasQ, DEKA M.E.L.A, Florence, Italy). The treatment protocol we used was as follows: spot size of 12 mm and fluence of 7 J/cm^2^. After two sessions of dye laser (at 45‐day intervals), the result was clinically optimal as shown in Figure 1. The skin was almost fully recovered, and no severe side effects were registered. An external cooling system (Burian PRO, DEKA M.E.L.A, Florence, Italy) was used during the sessions and a healing and soothing cream with hyaluronic acid was applied after the treatment.

**FIGURE 1:**
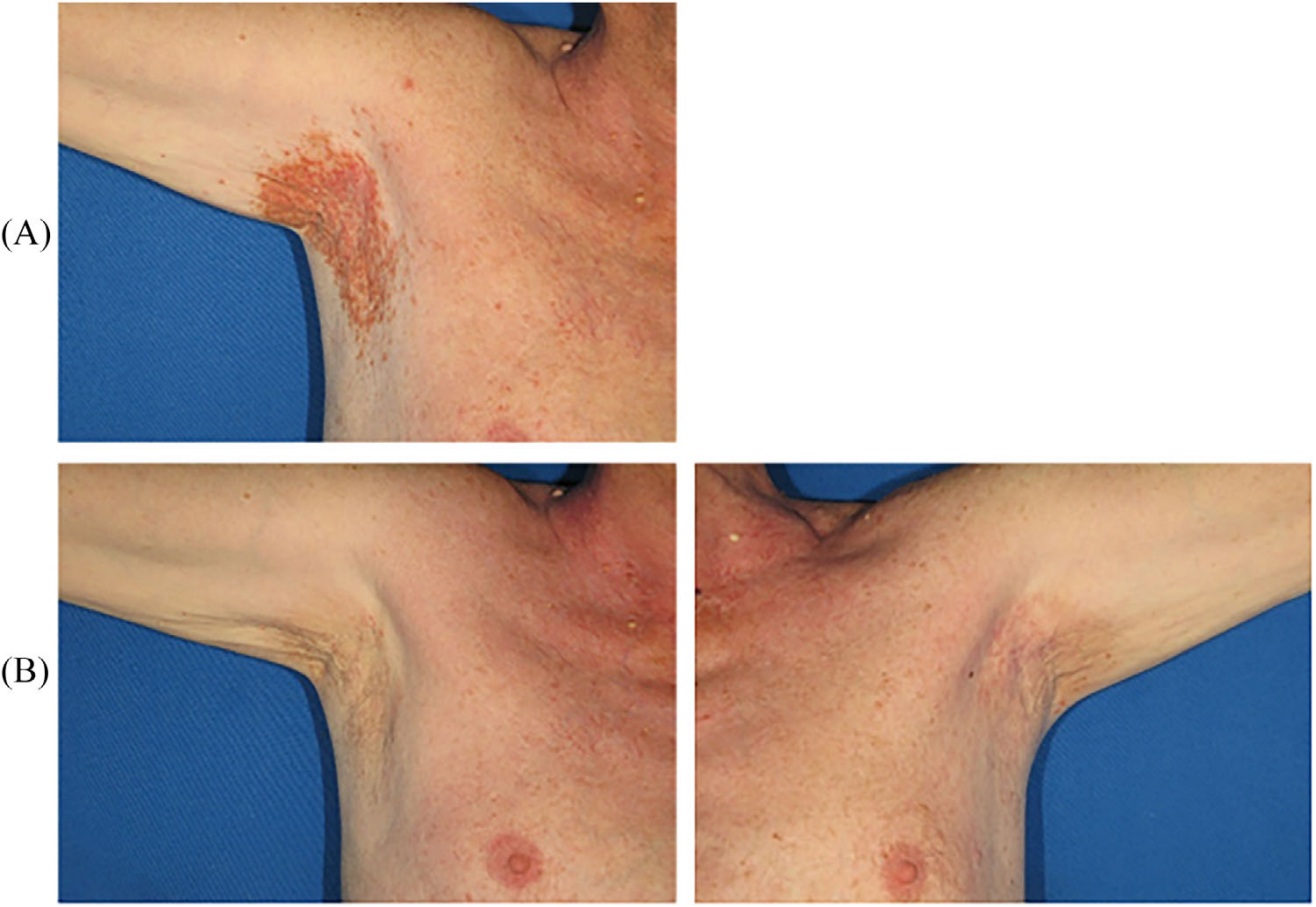
Clinical pictures of a male case of Darier disease patient. The axillary area was treated with two sessions of dye laser (at 45‐day intervals). (A) Baseline, before treatment. (B) Clinical results after two sessions of the dye laser. The picture was taken 2 months after the last laser session.

PDL is utilized to treat vascular lesions due to its high oxyhemoglobin absorption [5], but it has also been used to treat a variety of inflammatory disorders. It can stimulate the cutaneous immunological response by causing mild capillary damage and edema of endothelial cells in the dermal connective tissue. This damage produces and releases cytokines and growth factors, ultimately leading to the stimulation of new collagen [6, 7, 8]. PDL's success in an inflammatory condition, such as DD, could be attributed to both the lesions' vascular components and its immunomodulatory abilities.

In conclusion, our results support the limited literature already existing [8] regarding the treatment of DD with dye lasers. Indeed, it proved to be a valuable option to guarantee a long‐term reduction of the symptoms.

## Ethics Statement

The article is in accordance with the Declaration of Helsinki on Ethical Principles for Medical Research involving human subjects. Ethical approval is not necessary as the study device is already CE marked since 2013.

## Consent

Informed consent was obtained from all subjects involved in the study.

## Conflicts of Interest

T.Z., B.M.P., and I.F. were employed by El.En. Group. The remaining authors declare that the research was conducted in the absence of any commercial or financial relationships that could be construed as a potential conflict of interest.

## Data Availability

The data that support the findings of this study are available from the corresponding author upon reasonable request.
